# Adenine at lower doses acts in the kidney as an aquaretic agent and prevents hyponatremia

**DOI:** 10.1007/s11302-025-10105-7

**Published:** 2025-08-12

**Authors:** Alaa Alghamdi, Charuhas V. Thakar, Hassane Amlal

**Affiliations:** 1 Departments of Internal Medicine, Division of Nephrology & Hypertension, College of Medicine, University of Cincinnati, 231 Albert Sabin Way, MSB, Room 6213, Cincinnati, OH 45267 USA; 2 Department of Pharmacology and Systems Physiology, College of Medicine, University of Cincinnati, Cincinnati, OH 45267 USA; 3 Department of Pharmacology, College of Clinical Pharmacy, Imam Abdulrahman Ben Faisal University, Dammam, Saudi Arabia

**Keywords:** Adenine, AQP2, Vasopressin resistance, Aquaresis, Hyponatremia

## Abstract

We have previously reported that adenine at high doses interferes with the vasopressin signaling pathway, causes massive diuresis and volume depletion, and ultimately leads to renal failure. In the present study, we examined the effects of adenine on renal salt and water handling in a time course and dose–response study in rats housed in metabolic cages and fed control or adenine-containing diet at 1500, 2000, 2500 mg/kg and euthanized after 1, 3, and 7 weeks. Adenine at 2000 and 2500 mg/kg caused early and significant polyuria, polydipsia, and decreased urine osmolality in a dose-dependent manner without significantly affecting food intake, blood volume, blood electrolyte levels, or acid–base composition. The impaired water balance resulted from the downregulation of apical water channel AQP2 in the outer and inner medulla but not in the cortex. Adenine did not alter electrolytes (Na^+^, K^+^, Cl^−^) excretion at these doses for up to 3 weeks. However, a slight but significant increase in salt excretion was observed in adenine-fed rats for 7 weeks, which correlates with a significant downregulation of NKCC2, mostly in rats fed 2500 mg/kg adenine. Adenine-fed rats exhibited a substantial resistance to vasopressin in response to water deprivation or vasopressin treatment. Lastly, 2500 mg/kg adenine prevented the development of hyponatremia in a rat experimental model of the syndrome of inappropriate secretion of antidiuretic hormone (SIADH). In conclusion, adenine acts as an aquaretic agent in the kidney at lower doses and during a short feeding period. It can be used as a vasopressin antagonist in conditions associated with hyponatremia.

## Introduction

Pharmacological doses of adenine (5000 to 7500 mg/kg) adenine feeding for several weeks has been used by many investigators to generate animal (rat and mouse) models of renal failure [1–4]. At physiologic doses, adenine is metabolized through adenine phosphoribosyl transferase to form adenosine monophosphate (AMP), which is the precursor of several adenine derivatives, including adenosine, inosine monophosphate (IMP), hypoxanthine, xanthine, and uric acid [5–7]. However, long-term feeding of high doses of adenine produces 2,8-dihydroxyadenine (DHA) through the xanthine oxidase pathway [8, 9]. DHA, a water-insoluble metabolite, forms crystals that are deposited in renal tubules and interstitial tissues and, in chronic settings, leads to tubulointerstitial tissue injury [10, 11]. This has been the conventionally accepted mechanism for adenine-induced renal failure. However, our recent study examining the time-course effect of adenine feeding at a high dose (5000 mg/kg) demonstrated that oral or subcutaneous administration of adenine to rats is associated with an immediate (24 h) impairment in the urinary concentrating ability of the kidney [12]. At this dose, adenine inhibited food intake and caused significant water and salt wasting with subsequent volume depletion within 1 week and before the onset of renal function decline, as judged by creatine clearance studies [12]. Polyuria and increased salt excretion correlate with the downregulation of collecting duct apical water channel AQP2 and the thick ascending limb apical NaCl cotransporter NKCC2 in the kidneys of adenine-fed rats [12]. Adenine-induced urinary concentrating defect was not corrected by exogenous arginine vasopressin, and it is correlated with reduced cAMP production in vivo and in vitro [12]. Interestingly, these effects were all, to some extent, prevented in rats fed with adenine diet supplement with high salt [12]. It was concluded that anorexia and fluid loss with subsequent massive volume depletion are the primary events that lead to impaired renal function. Perhaps adenine-induced blood volume depletion and reduced renal function create an environment whereby DHA is insoluble and forms crystal deposits in the renal tubules and interstitial tissues [10, 11]. Based on our study, the mechanism by which adenine alters salt and water transport in the thick ascending limb and collecting duct appears to involve adenine interference with the vasopressin/V2 receptor signaling pathway. In this regard, biochemical and physiological studies have established adenine as a new signaling molecule, which acts via specific membrane-bound receptors expressed in many organs, including the kidney [12–15]. Adenine receptors belong to a family of G protein-coupled receptors that are linked to the inhibitory G protein (G_i_) and inhibit adenylate cyclase activity when stimulated with adenine, which leads to the reduction in intracellular cAMP production in brain cells as well as renal inner medullary collecting duct cells [7, 12–15]. We have previously demonstrated that lower doses of adenine specifically target water transport and impair the urinary concentrating ability of the kidney without affecting food intake [12]. This finding suggests that the effects of adenine on various physiological processes are likely dose-dependent. Hence, we hypothesized that the impact of lower doses of adenine could be limited to increased renal loss of salt and/or water, without changes in electrolyte intake, and likely without significant changes in blood volume status or renal function.

Thus, the objective of the following experiments was to re-examine the dose-dependent effects of adenine on renal water and salt transport in a time-course study in rats. The results demonstrate that lower doses of adenine feeding cause a significant impairment in water balance, which is correlated with the downregulation of AQP2 in the outer and inner medulla of the kidney without affecting salt excretion for at least up to 3 weeks. By 7 weeks, lower doses of adenine feeding are associated with a slight but significant increase in salt excretion that is correlated with downregulation of NKCC2 but not NHE3 in the kidney outer medulla. Lower doses of adenine did not alter food intake or body weight and did not cause massive volume depletion even after 7 weeks of adenine feeding. We further demonstrated that lower doses of adenine feeding are associated with vasopressin-resistant urinary concentrating defect and that a lower dose of adenine prevented the development of hyponatremia in a rat experimental model of the syndrome of inappropriate secretion of antidiuretic hormone or SIADH.

## Methods

Male Sprague Dawley rats (250–350 g) were used following an animal protocol that was approved by the Institutional Animal Care and Use Committee of the University of Cincinnati. Rats were purchased from Envigo (Indianapolis, IN, USA), housed two per cage with free access to rodent chow and distilled water, and maintained in a temperature-controlled room regulated on a 12-h light/dark cycle for 1 week before and during the following treatments.

### Animal treatments

#### Dose–response and time course of adenine feeding study

Male Sprague Dawley rats were placed individually in metabolic cages and allowed free access to rodent powdered chow and water as previously used and described in our laboratory [12, 16–18]. After 3 days of adjustment, control groups remained on powdered chow, whereas adenine groups received powdered chow supplemented with one of the following doses of adenine (1500, 2000, 2500 mg/kg). All rats (n = 4 to 5 rats/group) had free access to distilled water, and fresh food was provided daily for up to 7 weeks. Physiological data, including body weight, food intake, water intake, urine volume, urine osmolality, and urinary electrolyte excretion, were measured daily for the first week and for the average of the last 2 days after 3 and 7 weeks of different doses of adenine and their corresponding controls. After each treatment period (1, 3, and 7 weeks), the animals were sacrificed for blood and kidney tissue collection. Another set of rats was given rodent powdered chow alone (Control, *n* = 5) or supplemented with a higher dose (5000 mg/kg) of adenine (*n* = 6) and euthanized after 7 weeks of treatment.

At the end of each treatment, the animals were euthanized, and blood was collected from the heart, and the kidneys were rapidly removed and placed in ice-cold Hank’s solution. The cortex, an inner stripe of the outer medulla, and the inner medulla were then dissected and snap-frozen in liquid nitrogen and stored at −80 °C freezer. Blood chemistry was assessed using an i-STAT1 analyzer with EC8 + cartridges (Abbott Laboratories, Abbott Park, IL), which measures electrolyte levels, acid–base composition, blood glucose level, as well as the levels of extracellular fluid volume markers (i.e., BUN, Hct, and Hb). Urine Na^+^, K^+^, and Cl^−^ were determined using the Easylyte Plus Na/K/Cl analyzer, and urine osmolality was measured using the Advanced Micro Osmometer (Model 3300), as previously used in our laboratory [12, 16–18]. Serum and urine creatinine levels were measured using a creatinine assay kit following the vendor's (Millipore Sigma) protocol, and as previously described (12).

#### Urinary concentrating ability test of control and 2500 mg/kg adenine-fed rats

##### Renal response to dehydration test

Water deprivation is the natural way to induce dehydration and increase vasopressin secretion in mammals. Toward this end, male rats were placed individually in metabolic cages with free access to control (*n* = 4) or 2500 mg/kg adenine-containing (*n* = 5) diets and distilled water, as described above. After 6 days, rats in both groups were deprived of water and had free access to their respective diets for 2 days. Urine volume and urine osmolality were measured daily before and after water deprivation.

##### Response to exogenous vasopressin test

Male Sprague Dawley rats were placed individually in metabolic cages with free access to control (*n* = 4) or 2500 mg/kg adenine-containing (*n* = 5) diets and distilled water as described above. After day 5, rats in both groups were subjected to a single subcutaneous injection of a vasopressin V2 receptor agonist [deamino-Cys^1^, D-Arg^8^]-Vasopressin or dDAVP at 3 µg/100 g body weight. Urine volume and urine osmolality were measured before and after dDAVP injection.

#### SIADH rat model to study the therapeutic role of adenine in hyponatremia

**Fig. 1:**
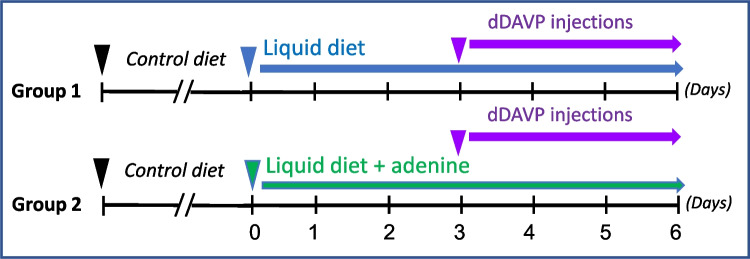
Study design diagram. Sprague Dawley rats were switched to a liquid diet prepared using rodent chow as described in Methods in the absence (*n* = 5) or presence (*n* = 5) of 2500 mg/kg adenine with free access to distilled water. After 3 days, rats in both groups were then injected daily with dDAVP (3 µg/100 g BW, SC.) and euthanized after 3 days. The consumption of a liquid diet allows the animals to be water-loaded while they satisfy their hunger and their needs in calorie intake

To address whether lower doses of adenine can antagonize vasopressin and prevent hypometria, we performed the following experiment as proof of concept. We used a modified version of the syndrome of inappropriate secretion of antidiuretic hormone (SIADH) protocol, initially described by Verbalis [19] and previously used by others [20–23]. Accordingly, rats were switched from solid rodent chow to a liquid diet composed of powdered rodent chow (25.5%, weight/volume), dextrose (7.5%, weight/volume), and distilled water (67%, volume/volume). A bottle of distilled water was also present in the metabolic cages. Rats were divided into two groups and treated as described in the schematic diagram depicted below (Fig. 1). **Group 1**: Rats were fed a liquid diet and after 3 days, they were injected daily with dDAVP (3 µg/100 g BW, SC.) for 3 additional days while consuming liquid diet to induce dilutional hyponatremia. **Group 2**: Rats were fed a liquid diet supplemented with 2500 mg/kg adenine for 3 days and then injected daily with dDAVP for an additional 3 days while consuming an adenine-containing liquid diet.

### Preparation of membrane fractions from the renal cortex, outer medulla, and inner medulla

The total cellular fraction containing plasma membrane and intracellular membrane proteins was prepared in the three kidney regions as previously described [16] and extensively used in our [12, 16–18]. Briefly, cortical, outer medullary, and inner medullary tissues from control and treated animals were homogenized in ice-cold isolation solution (250 mM sucrose and 10 mM triethanolamine, pH 7.6) containing protease inhibitors (phenazine methylsulfonyl fluoride, 0.1 mg/ml; leupeptin, 1 μg/ml) using a Polytron homogenizer. The homogenates were centrifuged at low speed (1,000 g) for 10 min at 4 °C, and resulting supernatants were spun at 100,000* g* for 90 min at 4 °C. The pellets containing plasma membrane and intracellular vesicles were suspended in an isolation solution with protease inhibitors. The total protein concentration was measured, and the membrane fractions were solubilized at 60 °C for 20 min in 1 × Laemmli buffer.

### Electrophoresis and immunoblotting

Immunoblotting experiments were carried out as previously described and used in our laboratory [12, 16–18]. Briefly, the solubilized membrane proteins were size fractionated on 8% (NKCC2 and NHE3) or 10% (AQP2) polyacrylamide mini gels (Novex, San Diego, CA) under denaturing conditions. The separated proteins were electrophoretically transferred to nitrocellulose membranes using a Bio-Rad transfer apparatus (Bio-Rad Laboratories, Hercules, CA). The membranes were first blocked with 5% milk proteins and then incubated overnight at 4 °C (cold room) with affinity-purified primary rabbit anti-AQP2, rabbit anti-NKCC2, or monoclonal anti-NHE3 antibodies. Actin or GAPDH was used as a control constitutive protein for the equity in protein loading in all gels. The membranes were washed and incubated with appropriate secondary antibodies. The sites of antigen–antibody complexation on the nitrocellulose membranes were visualized by using the chemiluminescence method (SuperSignal Substrate, Pierce), and the image was captured using the Kuik Quant Imager (www.kindlebio.com). The band density for each protein was quantitated by densitometric analysis (ImageQuant 5.0, Molecular Dynamics, Sunnyvale, CA) and expressed as a percentage of control.

### Materials

Paper blotting and nitrocellulose membranes were purchased from Midwest Scientific (St Louis, MO, USA). Actin and NHE3 antibodies were purchased from Santa Cruz biotechnology (Santa Cruz, Dallas, TX). AQP2 and NKCC2 were generated using commercial services (1, 2). Secondary antibodies, donkey anti-rabbit and mouse anti-goat, were purchased from Thermo Scientific (Rockford, IL) and Santa Cruz Biotechnology. Actin or GAPDH (Santa Cruz Biotechnology, Paso Robles, CA). Adenine, dDAVP, and all other chemicals were purchased from Millipore-Sigma Chemical (St Louis, MO, USA).

### Statistical analysis

Semi-quantification of Immunoblots was determined by densitometry using UN-SCAN-IT gel densitometry software (Silk Scientific Inc., Orem, UT, USA). The physiologic data are expressed as absolute values, and the densitometry of protein bands is expressed as % of control. The results are presented as means ± SE. Statistical significance between control and experimental groups was determined by one-way ANOVA or Student’s unpaired *t*-test as needed using SigmaPlot 13.0 software. *P* < 0.05 was considered significant.

## Results

### Actual doses of adenine ingested by the animals

Table 1 presents the actual doses of adenine ingested by the animals, calculated based on their food consumption at each dietary adenine level. As expected, adenine intake increased proportionally with the amount provided in the food. However, over time, adenine ingested declined due to reduced food consumption, particularly at the 2500 mg/kg dose, and was also influenced by changes in both food intake and body weight at the 2000 mg/kg dose after 7 weeks.

**Table 1:** Time course of the actual adenine ingested, as a function of the amount of adenine provided in food and expressed as mg/kg body weight

	1500 mg/kg	2000 mg/kg	2500 mg/kg
Week 1	94 ± 2.0	128 ± 4.8*	180 ± 5.7
Week 3	87 ± 3.2	118 ± 2.5*	155 ± 5.3
Week 7	80 ± 3.9^a^	105 ± 3.3^¥bc^	134 ± 14^§b^

*N* = 5 in each group. *P < 0.001 vs. 1500 mg/kg; ¶*P* < 0.001 vs. 2000 mg/kg; §*P* < 0.003 vs. 1500 mg/kg;¥*P* < 0.01 vs. 1500 mg/kg, ^a^*P* < 0.03 vs 1 week; ^b^*P* < 0.01 vs. 1 week; ^c^*P* < 0.05 vs. 3 weeks

### Blood composition of control and adenine-fed rats

Blood chemistry results depicted in Table 2 show that rats fed lower doses of adenine (1500 to 2500 mg/kg) for 7 weeks have a normal blood electrolyte profile, normal glucose, Hct, and HB levels, and unchanged acid–base composition, as compared to the control (Table 2). However, a significant increase in BUN and creatinine levels was observed in rats fed 2500 mg/kg adenine for 7 weeks (Table 2). As expected, rats fed a higher dose (5000 mg/kg) of adenine for 7 weeks showed a significant disorder in almost all measured blood components compared to both control and lower doses of adenine (Table 2). These effects result from the development of renal failure initiated by massive volume depletion and reduced food intake, as previously described in our laboratory [12].

**Table 2:** Blood composition of control and adenine-fed rats for 7 weeks

	N	N^a+^ (mM)	K^+^ (mM)	Cl^−^ (mM)	HCO_3_^−^ (mM)	pH (Ph unit)	Glucose (mg/dl)	BUN (mg/dl)	%Hct (PCV)	Hb (g/dl)	Cr (μmol/L)
Control	10	138 ± 0.43	4.53 ± 0.11	98 ± 0.43	32 ± 0.38	7.39 ± 0.01	187 ± 2.61	19 ± 0.59	39 ± 0.49	13 ± 0.17	24 ± 0.74
1500 mg/kg	5	139 ± 0.86	4.68 ± 0.12	98 ± 0.51	34 ± 0.73	7.41 ± 0.05	180 ± 9.20	20 ± 1.18	39 ± 0.75	13 ± 0.26	27 ± 1.23
2000 mg/kg	5	137 ± 0.58	4.83 ± 0.19	98 ± 0.73	31 ± 0.69	7.33 ± 0.03	175 ± 10.0	21 ± 1.59	39 ± 0.87	13 ± 0.30	27 ± 2.97
2500 mg/kg	5	138 ± 0.93	4.40 ± 0.17	97 ± 0.32	31 ± 0.31	7.36 ± 0.00	184 ± 4.45	28 ± 4.48^§^	38 ± 0.63	13 ± 0.22	33 ± 1.58^€Ω^
5000 mg/kg	6	130 ± 1.32^*^	5.42 ± 0.25^¥§^	91 ± 1.39*	27 ± 1.95^¶¥^	7.26 ± 0.02^≠¶^	159 ± 8.17	123 ± 4.22*	28 ± 1.69*	9 ± 0.58*	ND

*BUN* blood urea nitrogen, *Hct* hematocrit, and *Hb* hemoglobin, *Cr* Creatinine. Data are mean ± SEM*N* = 5 rats in each group

*N* = 5 rats in each group ;*P < 0.001 vs. each of other dose; ¥*P* < 0.02 vs. 2500 mg/kg; §*P* < 0.02 vs. Control; ¶*P* < 0.002 vs. 0.15%; ≠ P = 0.05 vs. Control. ^€^*P* < 0.001 vs. control, ^Ω^*P* < 0.01 vs. 1500 and 2000 mg/kg. *ND* Not determined

### Time course effects of one week of adenine feeding on food intake, water balance, and urine osmolality

**Fig. 2:**
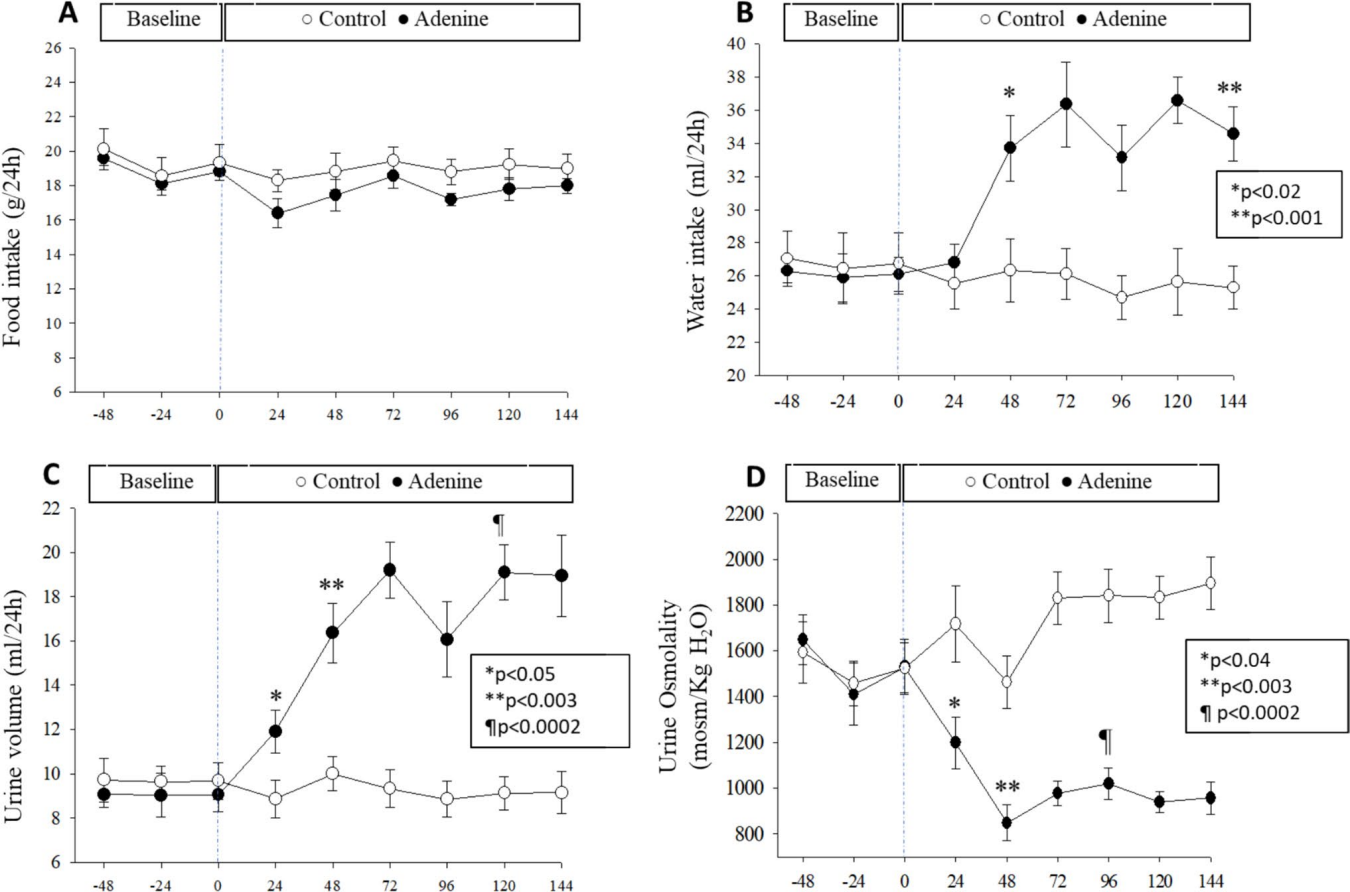
Low dose of Adenine alters water balance and urine osmolality without affecting food intake. Rats were placed individually in metabolic cages and had free access to powdered rodent chow in the absence (Control, *n* = 4) or presence of 2500 mg/kg adenine (*n* = 4) with free access to distilled water. **A** Food intake was not significantly affected by adenine for the duration of the experiment (P > 0.05 vs. Control. **B** Daily water intake in control and adenine-containing rodent chow. After switching to an adenine diet, water intake increased significantly within 48 h (*P,0.02, *n* = 4) and remained elevated for the duration of adenine feeding (**P < 0.001, *n* = 4), as compared to Control(*n* = 4). **C** Corresponding daily urine output, which increased significantly during the first 24 h after switching to adenine diet (*P < 0.05, **P < 0.003; *n* = 4) but increased incrementally after that to a maximum level after 6 days (¶*P* < 0.0002, *n* = 4) of the treatment, vs. Control (*n* = 4). **D** Urine osmolality dropped sharply within 24 h (*P < 0.04, *n* = 4) after switching to adenine diet, dropped further after 48 h (*P* < 0.003, *n* = 4), and remained low for the duration of the experiment (*P < 0.0002, *n* = 4) vs. Control (*n* = 4). These physiologic parameters remained unchanged during the duration of the experiment in rats fed a control diet. Daily data points are mean ± SEM. Data points at time zero are the average of 2 data points for a 2-day baseline period

Figure 2 is a time course study of the effects of adenine at 2500 mg/kg for 6 days on these physiological parameters. As shown, 2500 mg/kg adenine feeding to rats did not alter food intake (Fig. 2A, *P *> 0.05) but caused a significant increase in water intake (Fig. 2B, *P* < 0.001, *n* = 4) and urine volume (Fig. 2C, *P* < 0.0002, *n* = 4) along a sharp reduction in urine osmolality (Fig. 2D, *P* < 0.0002, *n* = 5) within the first 24 h, as compared to control (*n* = 4). Water intake (Fig. 2B) and urine volume (Fig. 2C) increased further and remained elevated for up to 6 days. Inversely, urine osmolality decreased further to a nadir value and remained low for the duration of the treatment (Fig. 2D).

### Time course and dose-dependent effect of adenine feeding on food intake, body weight, water balance, and urine osmolality

**Fig. 3:**
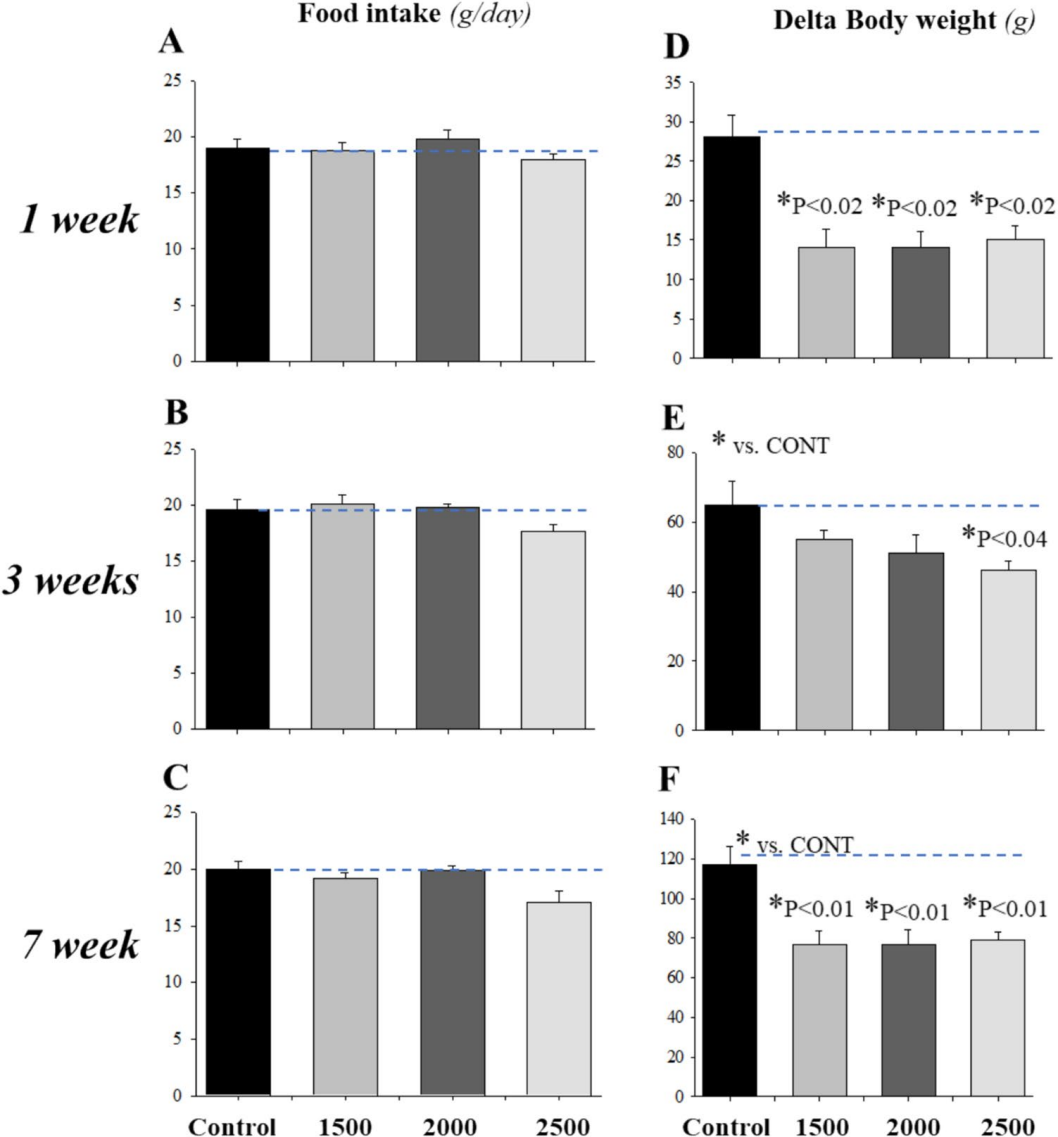
Dose–response and time course effects of adenine on food intake and body weight. Sprague Dawley Rats (*n* = 5) were placed individually in metabolic cages and fed control or adenine-containing diet at different doses with free access to distilled water. **A**: Adenine feeding did not affect food intake at any of the doses (*n* = 4/dose) tested vs. pooled controls (*P* > 0.05, *n* = 12). **B**: Adenine at all doses tested decreased body weight after 1 (*P* < 0.02, *n* = 4/dose) and 7 (*P* < 0.01, *n* = 4/dose) weeks feeding with some recovery at 3 weeks for 1500 and 2000 mg/kg adenine (*P* > 0.05, *n* = 4/dose), as compared to pooled controls (*n* = 12). The data presented are from the last day of control or adenine feeding

We have previously shown that a higher dose of adenine feeding (5000 mg/kg) to rats is associated with an early and sustained reduction in food intake with significant body weight loss within 1 week of adenine feeding [12]. Here, we demonstrate that lower doses of adenine (1500, 2000, and 2500 mg/kg) feeding to rats for up to 7 weeks did not significantly affect daily food intake (*P* > 0.05, *n* = 4, Fig. 3A, B, C) compared to control. However, adenine did lower body weight at all doses within the first week (*P* < 0.02, *n *= 4/group, Fig. 3D and F) and the body weight remained significantly low for up to 7 weeks (*P* < 0.01, *n* = 4/group, Fig. 3D and F) although some recovery of body weight was observed at the third week for 1500 and 2000 mg/kg adenine feeding (Fig. 3E), as compared to control.

**Fig. 4:**
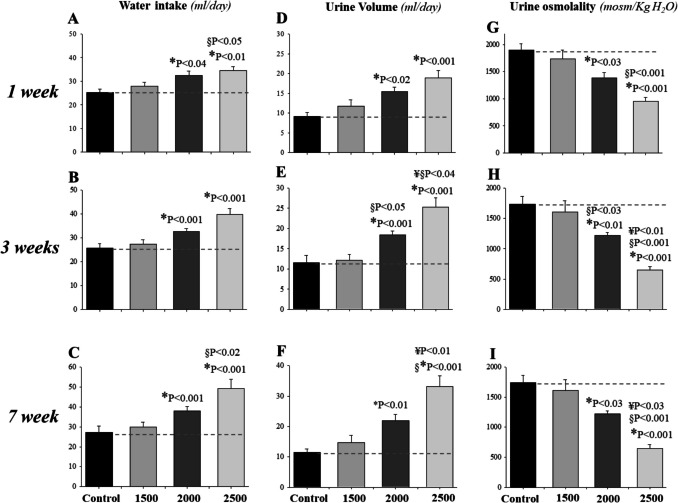
Dose–response and time course effects of adenine on water balance and urine osmolality. Rats were placed individually in metabolic cages with free access to distilled water and control or adenine-supplemented diet at 1500, 02000, or 2500 mg/kg and euthanized after 1 (n = 4/dose), 3 (*n* = 4/dose), or 7 (*n* = 4 to 5) weeks of treatment. As shown, adenine at 1500 mg/kg did not affect water intake (**A**, **B**, **C**), urine output (**D**, **E**, **F**), or urine osmolality (**G**, **H**, **I**) for any of the durations of the feeding (*P* > 0.05). However, adenine at 2000 and 2500 mg/kg significantly increased water intake (**A**, **B**, **C**) and urine volume (**D**, **E**, **F**) and reduced urine osmolality (**G**, **H**, **I**) in a time- and dose-dependent manner, as compared to pooled controls (*n* = 12). The data presented are from the last day of control or adenine feeding. (*) vs. Control; (§) vs. 1500 mg/kg adenine; (¥) vs. 2000 mg/kg adenine

Adenine feeding at lower dose of 1500 mg/kg to rats did not alter water balance or urine osmolality for the entire duration of the treatment, as compared to control (*P* > 0.05, *n* = 4, Fig. 4). However, a significant dose-dependent increase in water intake (*P* < 0.04 and *P* < 0.01, *n* = 4/group, Fig. 4A, B, C) and urine volume (*P* < 0.02 and *P* < 0.001, *n* = 4/group, Fig. 4D, E, F) with a parallel reduction in urine osmolality (*P* < 0.03 and *P* < 0.001, *n* = 4/group, Fig. 4G, H, I) were observed in response to adenine feeding at 2000 and 2500 mg/kg within the first week and remained as such for up to the seventh week of the treatment, as compared to control. Of note, the effect on these parameters is more profound with 2500 mg/kg adenine feeding, as compared to control or to the other lower doses.

### Time course and dose-dependent effect of adenine feeding on AQP2 protein abundance in the kidney

To determine the molecular mechanism underlying polyuria, polydipsia, and reduced urine osmolality in adenine-fed rats, we examined the protein abundance of the collecting duct apical water channel AQP2 in different regions of the kidneys harvested from rats fed different doses of adenine or a control diet for up to 7 weeks.

### AQP2 protein abundance in the kidney after 1 week of adenine feeding at 0.15%

As shown in Fig. 4, adenine feeding at a lower dose of 1500 mg/kg did not alter water intake, urine volume, or urine osmolality at any treatment period (Figs. 4A to I). As expected, AQP2 protein abundance was not changed by 1500 mg/kg adenine for any feeding duration in the kidney regions (Data not shown).

### AQP2 protein abundance in the kidney after 1 week of adenine feeding at 2000 and 2500 mg/kg

**Fig. 5:**
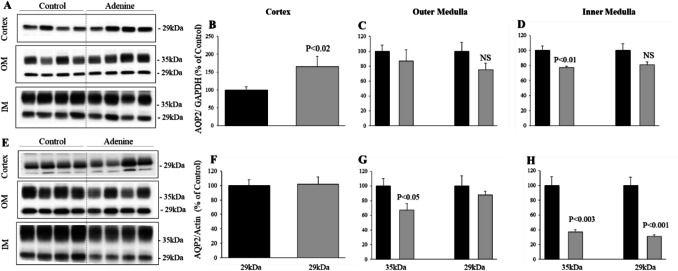
Expression of AQP2 protein in the kidneys of control and adenine-fed rats for 1 week. **A**, **E**: Immunoblotting of water channel AQP2 protein using membrane protein fractions isolated from cortex, outer medulla (OM) and inner medulla (IM) dissected from kidneys of rats fed control (*n* = 4) or adenine containing diet at 2000 mg/kg (**A**, *n* = 4) or 2500 mg/kg (**E**, *n* = 4) for 1 week. Right bar graphs are the corresponding average ± SEMs of the densitometry analysis of AQP2/actin or AQP2/GAPDH bands of control (dark bars) and adenine-fed rats (gray bars) at 2000 mg/kg (**B**, **C**, **D**) and 2500 mg/kg (**F**, **G**, **H**). At 2000 mg/kg, adenine downregulated the glycosylated form (35 kDa, *P *< 0.01, **D**) of AQP2 protein in the OM vs. Control. Interestingly, adenine at 2000 mg/kg increased AQP2 protein (*P* < 0.02, **B**) in the cortex and did not affect the abundance of AQP2 native band (29 kDa) in the IM vs. Control (**D**, *n* = 4 in each). At 2500 mg/kg, adenine decreased the abundance of AQP2 glycosylated (*P* < 0.05, **G**), but did not affect the native band (**G**, *P* > 0.05) in the OM. However, adenine at 2500 mg/kg downregulated both the glycosylated (35 kDa, *P* < 0.003, **H**) and native form (29 kDa, *P* < 0.001, **H**) in the IM vs. control. Each lane was loaded with 40, 20 and 3 μg of membrane proteins from cortex, OM, and IM, respectively, from different rats

After 1 week of 2000 mg/kg adenine feeding, AQP2 protein was not sharply altered throughout the kidney regions. In fact, AQP2 protein abundance is somewhat increased in the cortex (Figs. 5A and B, *P*< 0.02), whereas its expression was not affected in the outer medulla (Figs. 5A and C), vs. control (*P* > 0.05, *n* = 4 in each group). However, a slight but significant reduction in the glycosylated 35 kDa form is observed in the inner medulla of 2000 mg/kg adenine-fed rats (Figs. 5A and D, *P*< 0.01) vs. control (*n* = 4 in each group). This indicates that the increased water intake (Fig. 4A, *P*< 0.04) and increased urine volume (Fig. 4D, *P*< 0.02) along with reduced urine osmolality (Fig. 4G, *P*< 0.03) observed in rats fed 2000 mg/kg adenine is likely due to the inhibition of AQP2 trafficking to the apical membrane, as a result of adenine inhibition of vasopressin-induced cAMP stimulation in the collecting duct principal cells, as previously demonstrated in our laboratory [12]. The inhibition of vasopressin-induced cAMP production in the thick ascending limb likely resulted in NKCC2 activity reduction, which led to the increased salt excretion in rats fed adenine at 1500 and 2000 mg/kg for 7 weeks (Table 4), as NKCC2 protein abundance was not significantly altered under these conditions (data not shown).

After 1 week of 2500 mg/kg adenine feeding, the abundance of AQP2 protein is not altered in the cortex (Figs. 5E and F, *P*> 0.05). Only the 35 kDa band was slightly but significantly reduced in the outer medulla (Figs. 5E and G, *P*< 0.05). In contrast, both the glycosylated (35 kDa) and native (29 kDa) bands of AQP2 protein are sharply reduced (*P* < 0.003 and *P* < 0.001, respectively) in the inner medulla of 2500 mg/kg adenine feeding vs. Control (Figs. 5E and 5H, *n*= 4 in each group). The downregulation of AQP2 in the inner medulla (~ 60%) accounts for the significant polyuria and polydipsia along with reduced urine osmolality exhibited by rats fed 2500 mg/kg adenine (Figs. 2, 4A, D, and G).

### AQP2 protein abundance in the kidney regions after 3 and 7 weeks of adenine feeding

In the cortex, AQP2 protein abundance was not significantly altered in response to 2000 or 2500 mg/kg of adenine feeding for 3 or 7 weeks, as compared to the control adenine-free diet (Table 3, *n* = 4 in each group). In the outer medulla, AQP2 protein abundance is significantly downregulated only in response to 2500 mg/kg adenine feeding for both 3 weeks (−35%, *P* < 0.04, *n* = 4) and 7 weeks (−57%, *P* < 0.001, *n* = 5), as compared to control diet (Table 3). In the inner medulla, the AQP2 protein abundance is significantly downregulated in response to both 2000 mg/kg (*P* < 0.04) and 2500 mg/kg (*P* < 0.001) adenine during both 3 (*n* = 4) and 7 weeks (*n* = 5) of adenine feeding vs. control diet (Table 3). The downregulation of AQP2 protein in the outer and inner medulla accounts for the increased urine volume and water intake along with reduced urine osmolality caused by 2000 and 2500 mg/kg adenine during both periods of 3 and 7 weeks of feeding (Figs. 4B to I).

**Table 3:** AQP2 protein abundance expressed as AQP2/actin or AQP2/GAPDH ratios in the kidney regions of control and adenine-fed rats for 3 and 7 weeks

*Time*	WEEK 3			WEEK 7		
*Groups*	Control	2000 mg/kg	2500 mg/kg	Control	2000 mg/kg	2500 mg/kg
CORTEX	100 ± 28	92 ± 24	135 ± 45	100 ± 7	106 ± 8	109 ± 6
Outer Medulla	100 ± 15	73 ± 15	65 ± 13^*^	100 ± 10	82 ± 10	43 ± 7^¶§^
Inner Medulla	100 ± 9	55 ± 14^*^	54 ± 13^*^	100 ± 7	50 ± 11^*^	25 ± 2^¶§^

Data are mean ± SEM

*N* = 4 in each group; *P < 0.04 vs. pooled Controls (*n* = 8); ¶*P* < 0.001 vs. 2000 mg/kg adenine; §*P* < *P* < 0.0001 vs. pooled Controls (*n* = 8)

### Response of rats fed 2500 mg/kg adenine or control diets to water deprivation and exogenous vasopressin treatment

**Fig. 6:**
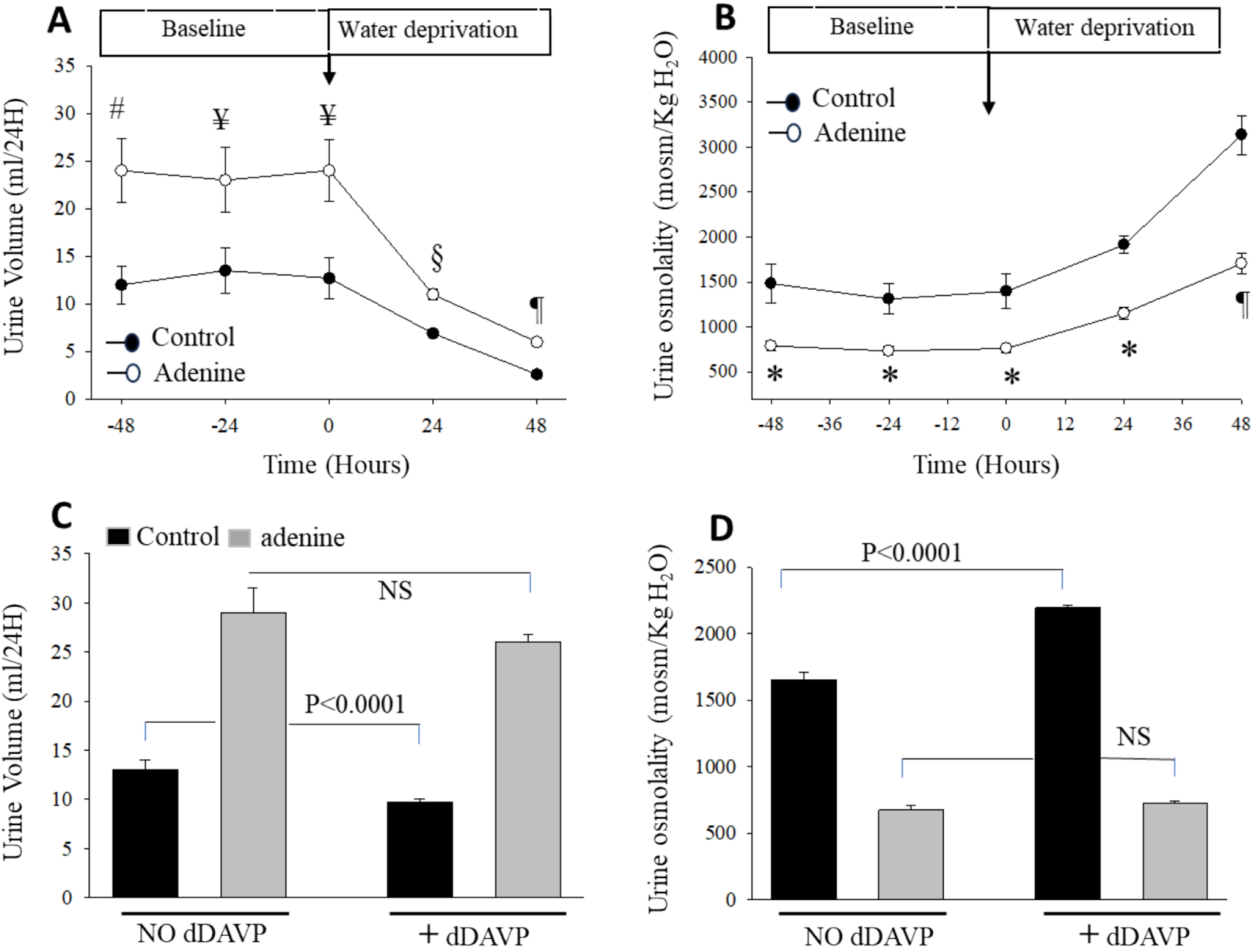
Adenine causes urinary concentrating defect and vasopressin resistance in rats. Rats were placed individually in metabolic cages and fed control or adenine (2500 mg/kg) containing diet for 6 days, and then both control and adenine-fed rats were deprived of water for 48 h. Urine volume (**A**) and urine osmolality (**B**) were measured daily before and after water deprivation. As shown, urine volume decreased sharply after water removal in control rats. However, adenine-fed rats exhibited a significantly higher water wasting at 24 (*P *< 0.002, *n* = 5) and 48 (*P *< 0.0005, *n* = 5) hours after water deprivation, as compared to control rats (*n* = 5). Urine osmolality increased with the same magnitude in the first 24 h but significantly less in adenine-fed rats during the second day (*P *< 0.0005, *n* = 5) of water deprivation, as compared to Control rats (*n* = 5). #*P *< 0.02, ¥*P *< 0.05, *P < 0.01, §*P *< 0.002, ¶*P *< 0.0005 vs. Control. Another set of rats was fed control or 2500 mg/kg adenine for 5 days and then injected with a single dose of vasopressin V2 receptor agonist dDAVP. Urine volume (**C**) and urine osmolality (**D**) were measured before and 24 h after dDAVP. In response to dDAVP, urine volume decreased significantly (*P *< 0.0001, *n* = 5) in Control but not (*P *> 0.05, *n* = 5) in adenine-fed rats, and urine osmolality increased significantly in Control (*P *< 0.0001, *n* = 5) but remained unchanged (*P *> 0.05, *n* = 5) in adenine-fed rats. NS: not significant

The kidney adapts to water deprivation by increasing water reabsorption through the upregulation of AQP2 in the collecting duct system. To test whether this function is conserved or impaired in rats fed 2500 mg/kg adenine, we subjected rats to water deprivation for up to 48 h. Accordingly, rats were fed a control or 2500 mg/kg adenine-containing diet for 6 days and then subjected to water deprivation for up to 48 h. As shown, 2500 mg/kg adenine feeding significantly increased urine output (Fig. 6A, *P*< 0.05) and decreased urine osmolality (Fig. 6B, *P*< 0.01) compared to the control diet (*n *= 5 in each group) at baseline. In response to water deprivation, urine output decreased sharply in both Control and adenine-fed rats. However, adenine-fed rats exhibited a significantly higher water loss after both 24 (Fig. 6A, *P*< 0.002), and 48 (Fig. 6A, *P*< 0.0005) hours, as compared to control rats (*n *= 5 in each group). In response to water deprivation, urine osmolality increased slightly with similar magnitude in both groups during the first 24 h (Fig. 6B). However, the increase in urine osmolality was sharply reduced in adenine-fed rats vs. controls during the 48 h period of water deprivation (Fig. 6B, *P *< 0.002, *n *= 5 in each group). This indicates that adenine at a lower dose of 2500 mg/kg causes a significant urinary concentrating defect.

To ascertain that adenine-induced urinary concentrating defect involves a renal resistance to vasopressin during dehydration, we tested the response of rats fed 2500 mg/kg adenine or control diets to exogenous V2 receptor agonist (dDAVP), as described in Methods. The results depicted in Fig. 6 show that dDAVP decreased urine output significantly in control (Fig. 6C, *P*< 0.00, *n* = 5) but not in adenine-fed rats (Fig. 6C, *P*> 0.05, *n* = 5). Similarly, dDAVP increased urine osmolality significantly in control (Fig. 6D, *P*< 0.0001, *n* = 5) but not in adenine-fed rats (Fig. 6D, *P*> 0.05, *n* = 5). These results suggest that a lower dose of adenine feeding causes a significant renal resistance to vasopressin and impairs the ability of the kidney to conserve water and concentrate urine in response to both dehydration and exogenous vasopressin treatment.

### Electrolytes excretion and NKCC2 expression: time course effects and adenine dose–response

**Fig. 7:**
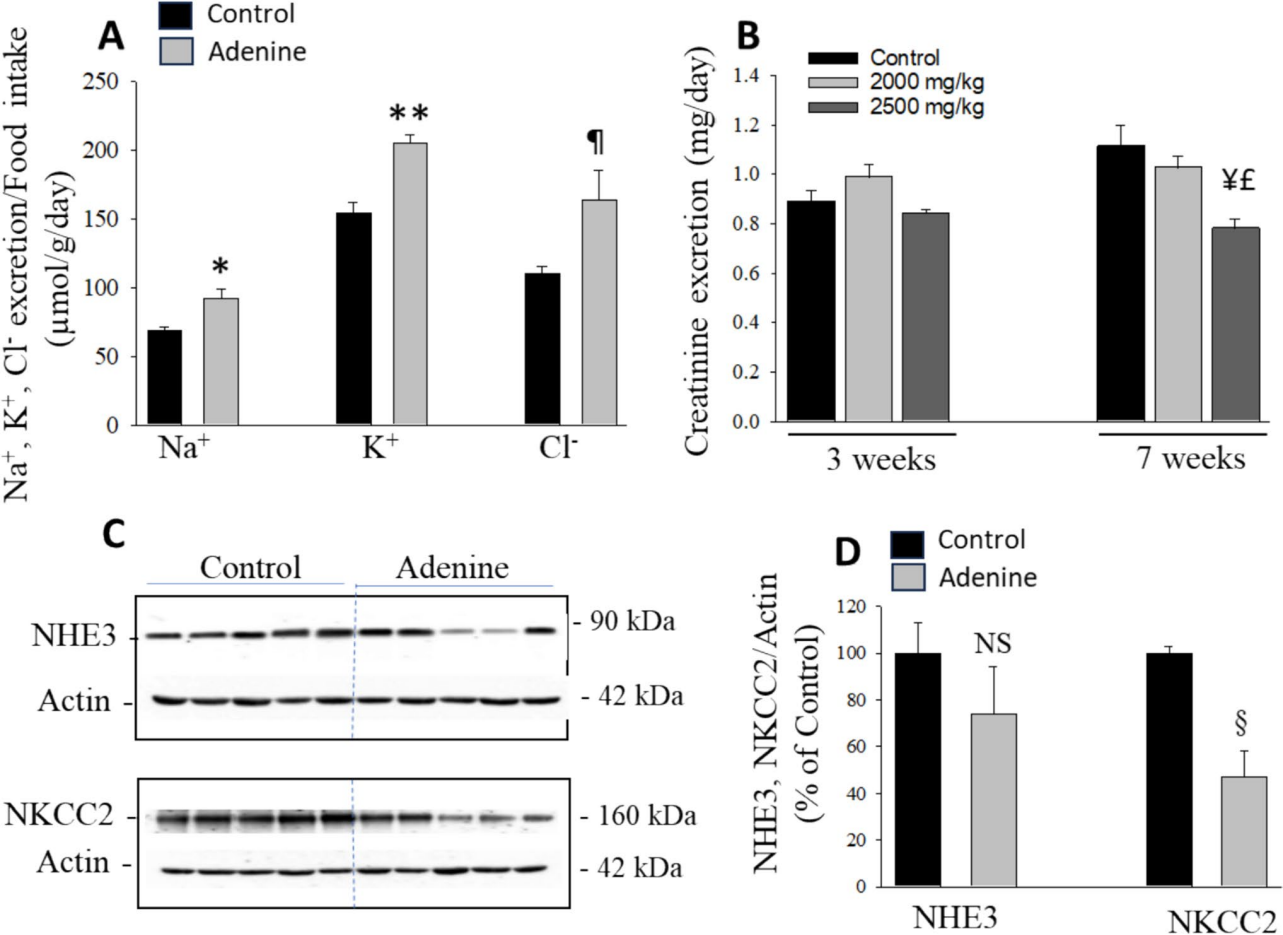
Electrolytes (Na^+^, K^+^, and Cl^−^) excretion, creatinine excretion, and expression of NKCC2 and NHE3 in the kidney outer medulla. Rats were fed a control or 2500 mg/kg adenine diet with free access to distilled water for 7 weeks. **A** Rats were placed individually in metabolic cages for food intake measurement and 24-h urine collection. Urinary Na^+^, K^+^, and Cl^−^ excretion was measured and adjusted for food intake in control vs. 2500 mg/kg adenine feeding. As shown, electrolyte excretion/food intake increase significantly in adenine-fed (*P < 0.02, **P < 0.001, and ¶*P* < 0.04, *n* = 5) vs. Control (*n* = 5) rats. **B** Urinary creatinine excretion in control and adenine-fed rats at 2000 or 2500 mg/kg for 3 or 7 weeks. A significant reduction (−30%) in creatinine excretion is observed only in rats fed adenine at 2500 mg/kg for 7 weeks. **C** Immunoblots of NHE3, NKCC2, and actin in membrane fractions isolated from the kidney outer medulla of Control and 2500 mg/kg adenine-fed rats for 7 weeks. **D**: Densitometry of NHE3 and NKCC2 normalized to actin. As shown, adenine feeding for 7 weeks significantly downregulated NKCC2 protein abundance (§*P* < 0.003, *n* = 5) but did not affect NHE3 protein expression (*P* > 0.05, *n* = 5) in the kidney outer medulla, as compared to Control (*n* = 5). Each lane was loaded with 20 μg (NHE3) or 10 μg (NKCC2) membrane proteins from the outer medulla of kidneys harvested from different rats. NS: Not significant

Urinary excretion of Na^+^, K^+^, and Cl^−^ was measured in urine collected during the last 24 h of 1, 3, and 7 weeks from rats fed control or adenine-containing diet at different doses (1500, 2000, or 2500 mg/kg). As shown in Table 4, the adenine diet, at all doses tested, did not affect the urinary excretion of Na^+^, K^+^, or Cl^−^ for up to 3 weeks, as compared to the control diet (Table 4, *P* > 0.05, *n* = 4 in each group). However, after long-term feeding (7 weeks), adenine did significantly increase the excretion of these electrolytes at 1500 mg/kg (Na^+^ and K^+^) and 2000 mg/kg (Na^+^, K^+^, Cl^−^) doses, as compared to the control diet (Table 4, *P* < 0.01, *n* = 4 in each). At 2500 mg/kg, adenine did not alter the absolute excretion of these electrolytes (Table 4, *P* > 0.05, *n* = 5 in each); however, at this dose adenine did reduce food intake and thus the electrolytes intake, and when the results are expressed as a ratio of electrolytes excretion over food intake, a significant wasting of Na^+^ (*P* < 0.02), K^+^ (*P* < 0.001) and Cl^−^ (*P* < 0.04) is observed in rats-fed 2500 mg/kg adenine vs. Control (Fig. 7A, *n* = 5 in each). The increase in Na^+^, K^+^, or Cl^−^ wasting in rat-fed adenine diet for 7 weeks (Table 4) likely results from the inhibition of NKCC2 activity at 1500 and 2000 mg/kg adenine feeding, as its protein abundance was not significantly altered (Data not shown), and from the downregulation of NKCC2 in response to 2500 mg/kg adenine, as demonstrated by the immunoblot depicted in Fig. 7C and corresponding densitometry scanning shown in Fig. 7D (*P* < 0.003 vs. Control, *n* = 5 in each). Adenine feeding at 2500 mg/kg did not significantly alter the expression of Na^+^/H^+^ exchanger NHE3 in the medullary thick ascending limb (P > 0.05, Figs. 7C and D), and similar results were observed for 1500 and 2000 mg/kg adenine feeding vs. control (data not shown).

**Table 4:** Time course and adenine (mg/kg) dose–response of urinary Na^+^, K^+^, and Cl^−^ excretion

*Time*	WEEK 1				WEEK 3				WEEK 7			
*Groups*	Control	1500	2000	2500	Control	1500	2000	2500	Control	1500	2000	2500
Na^+^ (mmol/day)	1.45 ± 0.14	1.62 ± 0.11	1.75 ± 0.06	1.57 ± 0.09	1.65 ± 0.12	1.73 ± 0.07	1.84 ± 0.07	1.69 ± 0.14	1.36 ± 0.06	2.00 ± 11^*^	2.18 ± 0.12^*^	1.56 ± 0.13
K + (mmol/day)	3.25 ± 0.20	3.69 ± 0.27	3.97 ± 0.13	3.39 ± 0.09	3.58 ± 0.25	3.71 ± 0.18	4.08 ± 0.14	3.72 ± 0.24	3.04 ± 0.18	4.01 ± 0.20^*^	4.39 ± 0.12^*^	3.44 ± 0.07
Cl^−^ (mmol/day)	2.92 ± 0.24	2.53 ± 0.18	2.87 ± 0.11	2.56 ± 0.09	2.61 ± 0.18	2.68 ± 0.09	3.03 ± 0.18	2.64 ± 0.15	2.18 ± 0.13	2.87 ± 0.16	3.35 ± 0.11^*^	2.73 ± 0.26

Data are mean ± SEM

*N* = 4 in each group.*P < 0.01 vs. pooled Controls (*n* = 4 to 5)

Figure 7B shows that adenine feeding at doses of 2000 and 2500 mg/kg for 3 weeks did not significantly alter creatinine excretion compared to control rats (*P* > 0.05, Fig. 7B; *n* = 5 per group). However, long-term adenine administration (7 weeks) at 2500 mg/kg resulted in a significant 30% reduction in creatinine excretion (*P* < 0.01, Fig. 7B; *n* = 5), while the 2000 mg/kg dose had no significant effect (*P *> 0.05, Fig. 7B; *n *= 5), relative to control. Creatinine excretion was consistent with serum creatinine levels, which increased significantly (~ 38%) after 7 weeks of adenine feeding at 2500 mg/kg compared to control (Table 2). In contrast, 3-week adenine feeding did not significantly affect serum creatinine levels at either 2000 mg/kg (27 ± 1.61 µmol/L, *n* = 5, *P* > 0.05) or 2500 mg/kg (25 ± 0.90 µmol/L, *n* = 5, *P* > 0.05) compared to control (25 ± 1.71 µmol/L, *n* = 5).

### Adenine at a lower dose prevents hyponatremia in a rat model of SIADH

**Fig. 8:**
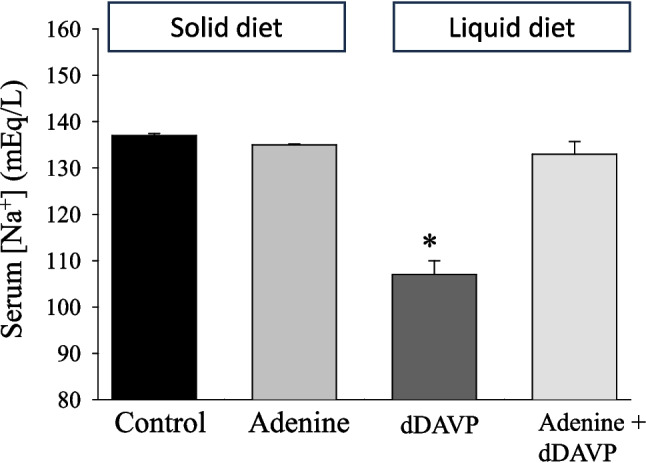
Adenine feeding prevents the development of hyponatremia in a rat model of SIADH. Serum [Na^+^] was measured in rats fed rodent chow alone (Control) or rodent chow supplemented with 2500 mg/kg adenine for 1 week. Another set of rats was fed a liquid diet alone (dDAVP) or a liquid diet supplemented with 2500 mg/kg adenine (Adenine + dDAVP) for 3 days, and then both groups were injected with dDAVP (3 µg/100 g, SC) for an additional 3 days. As shown, liquid diet feeding + dDAVP caused a significant reduction in serum [Na.^+^] or hyponatremia (*P < 0.001, *n* = 5), as compared to Control (*n* = 4) or adenine feeding alone (*n* = 4). Hyponatremia is prevented in the presence of 2500 mg/kg adenine (*P* > 0.05, *n* = 5) vs. Control (*n* = 4) or adenine feeding alone (*n* = 4)

We have previously shown that adenine inhibits vasopressin-induced cAMP production in vitro and urinary cAMP excretion in rats [12]. The above studies clearly demonstrate that adenine feeding at lower doses of 2000 and 2500 mg/kg in rats causes salt-free water wasting (aquaresis), at least for up to 3 weeks of treatment. Hence, the rationale behind this experiment is to test whether adenine can prevent the development of hyponatremia in a rat experimental model of SIADH. Indeed, the results depicted in Fig. 8 show that rats fed a liquid diet and treated with daily injections of dDAVP developed significant dilutional hyponatremia, as shown by a sharp reduction in serum [Na^+^] (Fig. 8, *P* < 0.001, *n* = 5), as compared to either control or 2500 mg/kg adenine-fed rats without dDAVP treatment. Interestingly, feeding rats with a liquid diet containing 2500 mg/kg adenine before daily injection of dDAVP prevented the development of hyponatremia (Fig. 8, *P* > 0.05, *n* = 5).

## Discussion

Adenine feeding at high doses (5000 and 7500 mg/kg) for several weeks is used extensively to develop an experimental rat model of chronic kidney disease (CKD) with subsequent death as a result of severe uremia [1–4, 24]. We have previously demonstrated that adenine causes nephrogenic diabetes insipidus with salt wasting, at least, by directly interfering with vasopressin V2 receptor signaling and subsequent downregulation of both NKCC2 and AQP2 in the kidney [12]. The impairment of water balance was observed within 2 days of adenine feeding, while renal function remained unchanged for up to 7 days [12]. In these studies, we have used a 5000 mg/kg adenine feeding protocol, which also causes a significant reduction of both food intake and body weight [12]. The combination of renal fluid loss and decreased food intake with subsequent massive volume depletion likely plays an important role in the initiation and progression of kidney injury in long-term high-dose adenine feeding [12]. Based on our results and prior studies [12–14, 25], it is concluded that adenine acts on renal tubules as a signaling molecule that inhibits vasopressin-stimulated cAMP through a membrane-bound G_i_ protein-coupled receptor. In this regard, it was obvious to stipulate that the effects of adenine on renal salt and water handling are likely dose-dependent. Indeed, the results of the present studies demonstrate that adenine feeding at lower doses of 2000 and 25,000 mg/kg in rats causes significant impairment in water balance (Fig. 4) without affecting salt excretion, at least for up to 3 weeks (Table 4). Blood composition data depicted in Table 2 demonstrate that adenine feeding to rats at 2000 and 25000 mg/kg for 7 weeks did not significantly alter blood electrolytes, hematocrit, hemoglobin levels, and did not alter serum K^+^ levels, or acid–base composition. In contrast, adenine feeding at a higher dose of 5000 mg/kg for 7 weeks causes a significant volume depletion with a sharp reduction in renal function, metabolic acidosis, and significant hyperkalemia (Table 2).

The increase in serum BUN and creatinine levels (Table 2) aligns with the observed reduction in creatinine excretion (Fig. 7B) in rats fed adenine at 2500 mg/kg for 7 weeks. These changes indicate a decrease in renal function, which appears to result from volume depletion. The latter is likely due to the downregulation of NKCC2 and AQP2 (Figs. 7C and D; Table 3), leading to excessive salt and water loss (Table 4; Figs. 4 and 7A). We have previously shown that a higher dose of 5000 mg/kg of adenine caused the downregulation of AQP2 and NKCC2, which resulted in significant salt and water wasting before the onset of renal failure (12). We should emphasize that the short-term effects of lower dose adenine (2000 and 25000 mg/kg) on renal function and renal tubular salt and water transport are specific to rat, as it has been established that the adenine dosage required for chronic kidney injury in rat and mice are different [reviewed in 26]. Rats are more resistant to adenine, and the dose of adenine needed to induce renal failure is set to 7500 mg/kg in rodent diets [26]. Conversely, mice are more sensitive to adenine because renal failure can be achieved by feeding mice 2000 and 25000 mg/kg adenine [26]. This difference in sensitivity to adenine between rats and mice likely results from the abundance and expression of adenine receptors in the kidney. Adenine is now established as a signaling molecule [7, 12, 14, 25] and was identified as the endogenous ligand of the Mas-related gene receptor A (MRGPRA), also called rat adenine receptor (rAdeR) [13]. In mice, however, two adenine receptors (mAde1R and mAde2R) have been characterized and were shown to be stimulated by nanomolar concentrations of adenine [27]. In rats, the adenine receptor is expressed mainly in the outer and inner medulla [14], indicating its expression in both the medullary thick ascending limb and collecting duct system, as exhibited by the downregulation of both NKCC2 and AQP2 in these segments of the nephron, respectively [12]. In mice, an immunolocalization study demonstrated that both mAde1R and mAde2R were expressed in the kidney; however, the antibody used in this study may not be very selective for one of the mAdeR, as the sequence of the immunogen peptide from rAdeR used to generate the antibody has 78% and 85% homology with mAde1R and mAde2R, respectively [15]. Additional studies are needed to determine the distribution of these receptors in mouse kidneys and study the effects of adenine on mouse renal tubular fluid handling in a dose-dependent manner. We should also mention that some of the effects results are likely the consequence of the hemodynamic and renal effects of the adenine overload rather than a direct effect of adenine acting on its receptor, specifically for long-term (7 weeks) feeding at 2500 mg/kg. Future studies will examine the pharmacokinetics of lower doses of adenine as well as their effects on the kidney structure, including both glomerular and tubular morphologies.

Nevertheless, adenine feeding to rats at lower doses of 2000 and 25,000 mg/kg and, at least, for up to 3 weeks is clearly associated with pure aquaresis without affecting salt excretion or blood volume. At these doses**,** adenine specifically targets AQP2 in the outer and inner medulla but not in the cortex (Fig. 5 and Table 3). Adenine-induced AQP2 downregulation in the outer and inner medullary collecting duct causes water wasting (polyuria), which is compensated for with an increase in water intake (polydipsia), allowing the animals to maintain a normal blood volume status (Table 2). The downregulation of AQP2 by a lower dose of 2500 mg/kg is due to the interference of adenine with the vasopressin signaling pathway in the collecting duct system. This was verified by the present experiments, which demonstrate a significant urinary concentrating defect in rats fed with 2500 mg/kg adenine diet and water-deprived for 48 h or treated with exogenous vasopressin (Fig. 6). This result agrees with our previous finding that adenine prevents urinary concentration in response to exogenous vasopressin treatment in 5000 mg/kg adenine-fed rats [12]. Hence, altogether, these results clearly indicate that lower doses (2000 and 25000 mg/kg) of adenine, at least for a short period of feeding, specifically target AQP2 in the outer and inner medullary collecting duct and increase electrolyte-free water excretion or aquaresis in rats. Future studies are needed to examine the pharmacokinetics and metabolic fate of adenine in rodents as well as in humans.

With regards to the clinical implications of the present study, our results show that the aquaretic dose of 2500 mg/kg adenine efficiently inhibited vasopressin-induced dilutional hyponatremia in an experimental rat model of SIADH (Fig. 8). Hyponatremia, the most common electrolyte abnormality in hospitalized patients [28], is usually linked to increased circulating levels of vasopressin and subsequent increase in water retention by the kidney in many conditions, including congestive heart failure, liver cirrhosis and SIADH [23, 29–34]. In this regard, tolvaptan, a nonpeptide vasopressin V2 receptor antagonist, inhibits water reabsorption in the collecting duct system and causes electrolyte-free water excretion by the kidney [35]. Tolvaptan was shown to decrease edema efficiently and correct hyponatremia in patients with heart failure [21, 36–40]. Moreover, recent studies provided strong evidence for the efficacy of tolvaptan in slowing kidney growth and renal function decline in autosomal polycystic kidney disease [41–43]. Similarly, our present studies demonstrate that lower doses of adenine can also interfere with vasopressin signaling by activating G_i_-protein-coupled adenine receptors and increasing water-free electrolyte excretion without changing blood volume or renal function (Tables 2 and 4). Hence, we propose that adenine, at lower doses and for a short time, can be used as a new line of treatment for water disorders, including the correction or prevention of hyponatremia.

In conclusion, at lower doses and for a short period, adenine acts in the rat kidney as an aquaretic agent that downregulates AQP2 in the outer and inner medullary collecting duct system. Adenine antagonizes the hydro-osmotic activity of vasopressin and inhibits water reabsorption without changing the total level of electrolyte excretion and without adversely affecting serum electrolytes, blood volume, or renal function. These studies pave the way for the use of adenine or its derivatives as a new line of treatment for hyponatremia.

## Data Availability

No datasets were generated or analysed during the current study.
